# Distribution patterns and drivers of urban green space and plant diversity in Haikou, China

**DOI:** 10.3389/fpls.2023.1202115

**Published:** 2023-08-21

**Authors:** Hai-Li Zhang, Lin-Yuan Guo, Mir Muhammad Nizamani, Hua-Feng Wang

**Affiliations:** ^1^Sanya Nanfan Research Institute of Hainan University, Hainan Yazhou Bay Seed Laboratory, Hainan University, Sanya, China; ^2^Collaborative Innovation Center of Nanfan and High-Efficiency Tropical Agriculture, School of Tropical Crops, Hainan University, Haikou, China; ^3^Department of Plant Pathology, Agricultural College, Guizhou University, Guiyang, China

**Keywords:** conservation, urban plant diversity (UPD), urban functional unit (UFU), maintenance times, watering frequency

## Abstract

Investigating historical and ongoing changes in urban green space (UGS) and urban plant diversity (UPD) provides critical insights into urban ecology and urban planning development. The present study illuminates some of the transformations which can occur in rapidly developing urban landscapes. In this work, we used 30 m resolution images from the Landsat 5 satellite from 2015 to investigate UGS patterns in Haikou City, China. Metrics of UPD were obtained using field surveys, allowing the proportion of UGS and UPD to be determined in each urban functional unit (UFU) of Haikou. The results revealed that leisure and entertainment areas (such as parks) had the highest diversity, whereas roads and transportation hubs had the lowest. More frequent anthropogenic maintenance had a positive effect on the total number of species, including cultivated, tree, and herb species. Similarly, increased watering frequency had a positive impact on the diversity of cultivated and shrub species. By providing demonstrating a crucial link between UGS and UPD, the results provide valuable information for planning sustainable urban development in Haikou City and other tropical regions. They highlight the important role of UGS in maintaining biodiversity and providing a range of ecosystem services. This research will inform policymakers and urban planners about the need to consider UGS and UPD in urban planning and management process, in order to promote sustainability and conservation of biodiversity.

## 1 Highlights

1. Leisure and entertainment areas have the highest proportion of urban green space, while transport areas have the lowest.
2. Leisure and entertainment areas have the highest cultivated, spontaneous, and total species richness.
3. Urban green spaces positively affect the number of spontaneous species and herb species.
4. Maintenance times positively affect the number of total species, cultivated species, tree species, and herb species.

## 2 Introduction

Understanding the intricate relationship between urban green space (UGS) and urban functional units (UFUs) is paramount for improving urban sustainability (Cheng et al., 2022). In general, UGS can be defined as vegetation-dominated urban land. Integrating UGS into the fabric of various UFUs can serve as a critical strategy for promoting urban biodiversity, improving air and water quality, and mitigating the effects of climate change (Felson and Pickett, 2005; Zhang et al., 2022a). For instance, incorporating green roofs and walls into residential and commercial UFUs has been shown to increase aesthetic appeal, create additional micro-habitats for urban flora and fauna, enhance thermal insulation, and reduce stormwater runoff (Ngan, 2004). Similarly, integrating vegetation into industrial UFUs can provide buffering against noise and pollutants, reducing their negative environmental impact on surrounding localities (Ramaiah and Avtar, 2019).

However, simply adding UGS to various UFUs does not guarantee success (Li et al., 2005). Successful integration requires a comprehensive understanding of the urban ecosystem, including socio-ecological dynamics (Niemelä et al., 2010). The preferences and behaviors of city residents, economic viability of green infrastructure, and local environmental conditions must all be considered (Thorne et al., 2018). Understanding these complex dynamics is essential for developing sustainable urban landscapes which can support both human well-being and biodiversity (Bennett et al., 2015). As one example, the design of UGS in residential areas should consider residents’ preferences for certain types of vegetation or recreational facilities, while also ensuring that suitable habitats are created for local fauna (Mathey et al., 2015; Harris et al., 2018). Likewise, the implementation of green infrastructure in industrial UFUs needs to account for any economic trade-offs or potential co-benefits, such as cost savings from reduced energy use, or improved public image from enhanced environmental performance.

The importance of UGS in promoting human well-being and sustaining biodiversity has gained increasing recognition in recent years (Chen et al., 2018; Zhang et al., 2022a). UGS provides numerous environmental benefits, including mitigating erosion and runoff, and reducing air, water, soil, and noise pollution (Leung et al., 2011; Ramaiah and Avtar, 2019). In addition, UGS has been shown to significantly improve urban livability (Benne and Mang, 2015; McPhearson et al., 2016; Li X. et al., 2021). Several strategies are commonly employed to increase UGS within cities, including planting street trees, developing parks and green spaces, planting vegetation alongside watercourses, and creating green buildings and rooftop gardens (Aronson et al., 2017; Li Q. et al., 2021). In China, the development of garden cities has become a major goal in many urban areas to promote a better aesthetic environment, facilitate progress toward climate goals, and realize the vision of “ecological civilization” – a type of civilization that maintains a sustainable and harmonious relationship between humans and nature (Liu et al., 2014).

The composition of UGS in urban areas can vary widely, ranging from homogeneous grassed lawns, monocultures, and non-native species, to more biodiverse urban parks and woodlands. While the concept of incorporating UGS into city planning has long been understood, the importance of species diversity has only been recently brought to the fore (Hunter et al., 2019). This is particularly important, as the diversity of animals in urban areas may be linked to the diversity of the plant life (Hughes et al., 2022; Nava-Diaz et al., 2022; Benedetti et al., 2023). Therefore, understanding influences on urban plant diversity (UPD) is crucial as a driver of overall urban biodiversity. UPD can be influenced by various factors, including the city’s UGS, management and history, and the size and distance to natural areas (Čeplová et al., 2017). Relatively few native plants remain in most urban environments; this is particularly true for mature native trees, which are almost inevitably removed during development. Consequently, the majority of plants within urban areas are planted and largely non-native. However, enhancing native biodiversity is particularly important in the management and planning of urban spaces (Rejmánek et al., 2013; Nizamani et al., 2021).

The distribution of UPD within an urban environment is related to the area of UGS, the amount and type of urban vegetation, and other management constraints (Ortí et al., 2022). With rapid urbanization occurring in many developing regions across the globe, it becomes crucial to understand how UPD and UGS can be maintained to preserve urban biodiversity (Zhang et al., 2022a). The dynamics of UGS and UPD are intricately linked to land-use alterations, socio-economic disparities, and urban management strategies. Intense urbanization often reduces UGS and UPD, while conscious green infrastructure planning can enhance UGS and UPD (Nizamani et al., 2021). Other mechanisms that can drive UPD and UGS include the area of green space, construction age, housing price, population density, and management approaches (Niemelä et al., 2010; Guo et al., 2018; Cheng et al., 2022). The extent of urban development also affects plant diversity, as areas with more high-rise buildings have fewer green spaces for plants to thrive (Kuussaari et al., 2021). Affluent neighborhoods typically harbor more green spaces; however, this correlation does vary regionally (Zhang et al., 2022b). Finally, purposeful management strategies prioritizing green space preservation and diversity – such as include selecting and planting street trees, managing extensive green areas, and designing green roofs – help promote UGS and UPD. On the other hand, neglect and mismanagement can cause their decline (Zhang et al., 2022a).

The focus of this study is Haikou, the political, cultural, and economic hub of Hainan Province. Although it is considered a biodiversity hotspot in China, it is undergoing rapid urbanization (Lin and Fu, 2022). Examining UGS and UPD in Haikou can help identify and conserve native or rare plant species, thus preserving their unique biodiversity. Haikou also has a rich cultural history, including traditional gardens and parks. Research on UGS and UPD can aid in conserving and restoring these cultural landscapes, promoting a sense of identity and pride among residents. By studying the interplay between UGS and UPD, researchers can better understand the ecosystem services these spaces provide and plant diversity. This knowledge allows for a more comprehensive understanding of how different UGS and UPD contribute to ecosystem restoration in Haikou, such as mitigating the urban heat island effect, reducing air pollution, and enhancing stormwater management.

This study aims to obtain data on the change of UGS in Haikou City using 30m Landsat satellite images from 2015, and the change of UPD using field survey data (Wang et al., 2020; Zhang et al., 2022a). The specific objectives were (1) to examine the proportion of UGS and UPD in each urban functional unit (UFU) in Haikou City in 2015 and (2) to assess the potential driving factors of UGS and UPD in Haikou City, including land use, socio-economic, and management factors.

## 3 Methods

### 3.1 Study area

**Figure 1:**
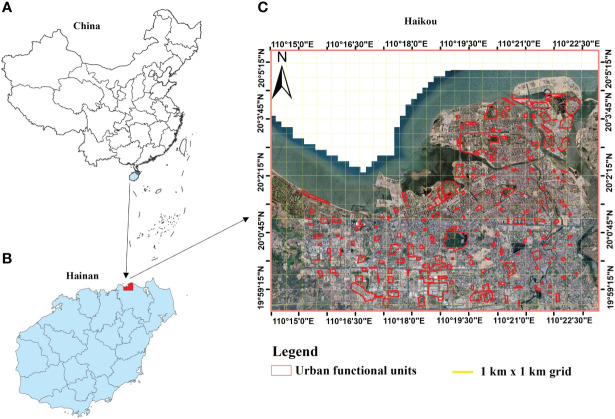
The geographic location of Haikou; **(A)** Map of China highlighting Hainan, **(B)** Map of Hainan highlighting Haikou City, and **(C)** Satellite map of Haikou City (https://www.google.com/maps) showing the urban functional units and plot samples (UFUs, red boundaries) surveyed in this study.

Haikou, the capital of Hainan Province, is an island city bordering the Qiongzhou Strait and 18 km from the south coast of the Chinese mainland (Zhang et al., 2022a). The study area was conducted in an urbanized region of Haikou, which ranges from 110°1’’ to 110°2’’ E and 19°32’’ to 20°0’’ N (**Figure 1**). It is believed that Haikou first became urbanized during the Han Dynasty, approximately 2,200 years ago (Swope, 2014).

### 3.2 Sampling design for measuring urban functional units

**Figure 2:**
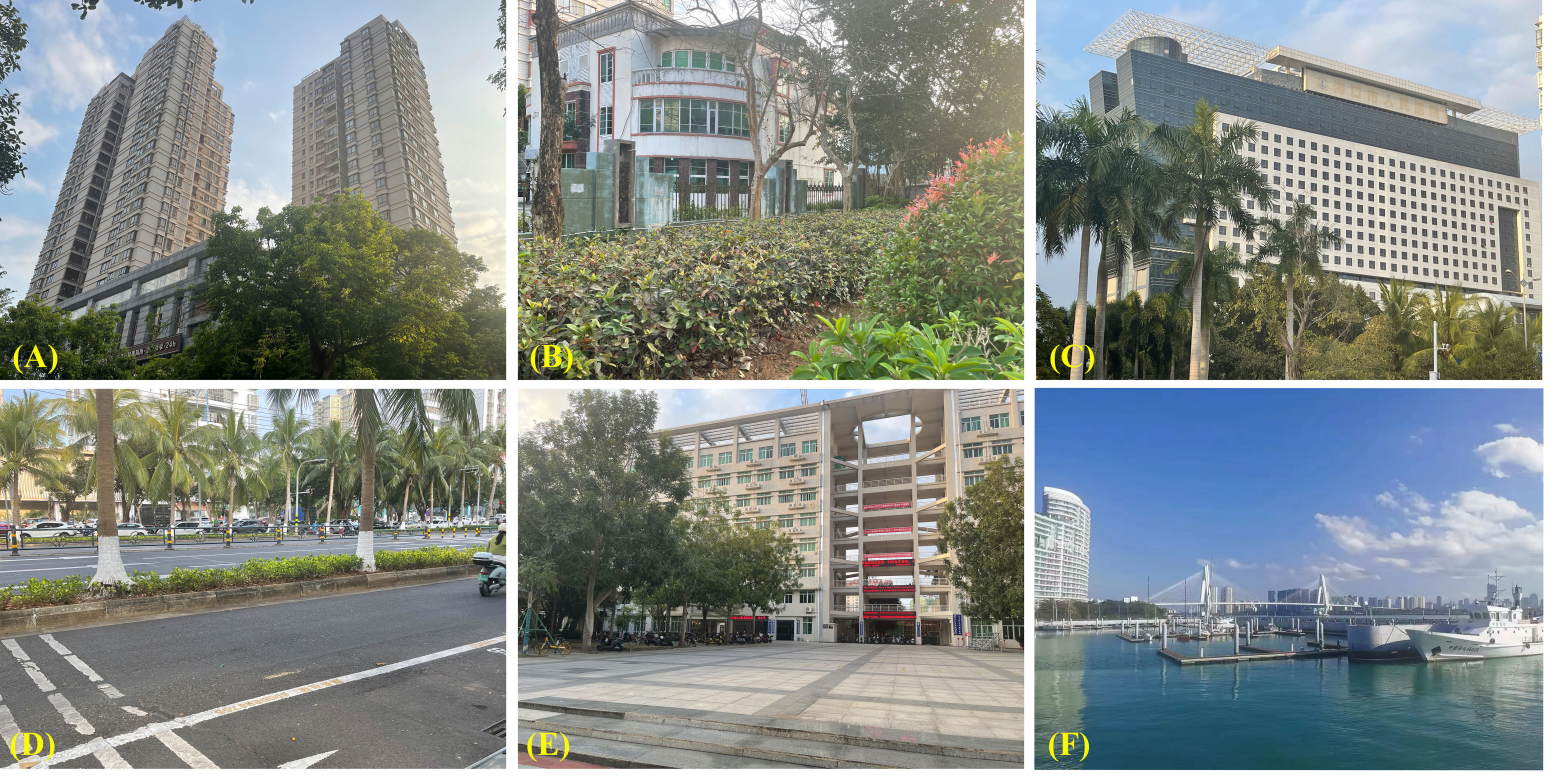
Example of secondary types of urban functional units of Haikou. **(A)** High-Density Residential Area. **(B)** Low-Density Residential Area. **(C)** Hospital. **(D)** Road. **(E)** Colleges/University. **(F)** Transportation hub.

To delineate the sampling plots for UGS and UPD measurements in Haikou, the main urban area was divided into 1 km × 1 km grids using Landsat 5 (https://www.usgs.gov/) remote sensing images (Wang et al., 2016; Nizamani et al., 2021; Zhang et al., 2022a). To ensure a representative distribution of the selected UFUs in Haikou City, we randomly selected at least one UFU within each 1 km × 1 km grid. UFUs were given a primary and secondary UFU classification based on the Urban Forest Effect (UFORE) model (UFORE, 2012) and the 2018 mapping of essential urban land use categories in China (EULUC-China) (Gong et al., 2020). Primary UFUs were broadly classified as institutional businesses, industrial and commercial areas, residential areas, leisure and entertainment areas, and transport areas (Wang et al., 2020; Cheng et al., 2022; Zhang et al., 2022a). Each primary UFU was divided into more detailed secondary UFUs, which included categories such as hospitals, primary and secondary schools, research institutes, universities, industrial areas, commercial areas, high-rise residential, low-rise residential, parks, square and sports centers, roads, and transportation hubs (**Figure 2**). After selecting the UFUs, a field survey was conducted to obtain UPD data. In total, 111 UFUs were identified with complete study data (**Figure 1C**; **Table 1**).

**Table 1:** The classification of the primary and secondary UFUs sampled in Haikou for this study.

Primary UFU	Number	Secondary UFU	Number
Institution business area	44	Hospital	6
		Government organization	8
		Primary and secondary schools	15
		Research institutes	7
		University	8
Industrial and commercial area	19	Industrial area	11
		Commercial area	8
Residential area	31	High-rise residential	26
		Low-rise residential	5
Leisure and entertainment area	7	Parks	6
		Square and sport center	1
Transport area	10	Road	8
		Transportation hub	2
Total	111		111

### 3.3 Survey of urban plant diversity

**Figure 3:**
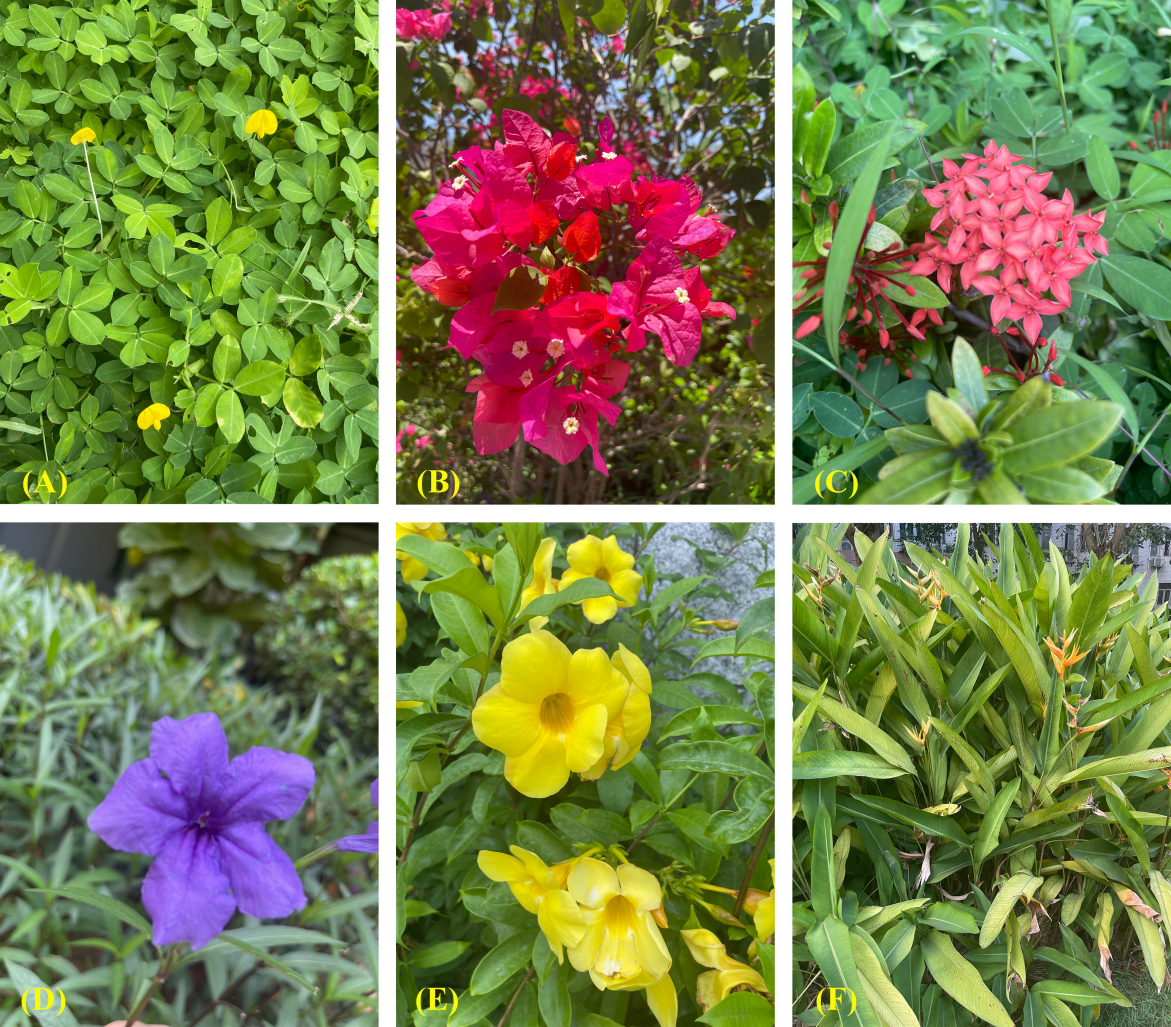
Examples of cultivated species occurring within secondary urban functional units. **(A)** *Arachis pintoi Krapov.* et W. **(C)** Greg. **(B)** *Bougainvillea spectabilis* Willd. **(C)** *Ixora chinensis* Lam. **(D)** *Ruellia simplex.* **(E)** *Allamanda cathartica* L. **(F)** *Strelitzia reginae* Aiton.

**Figure 4:**
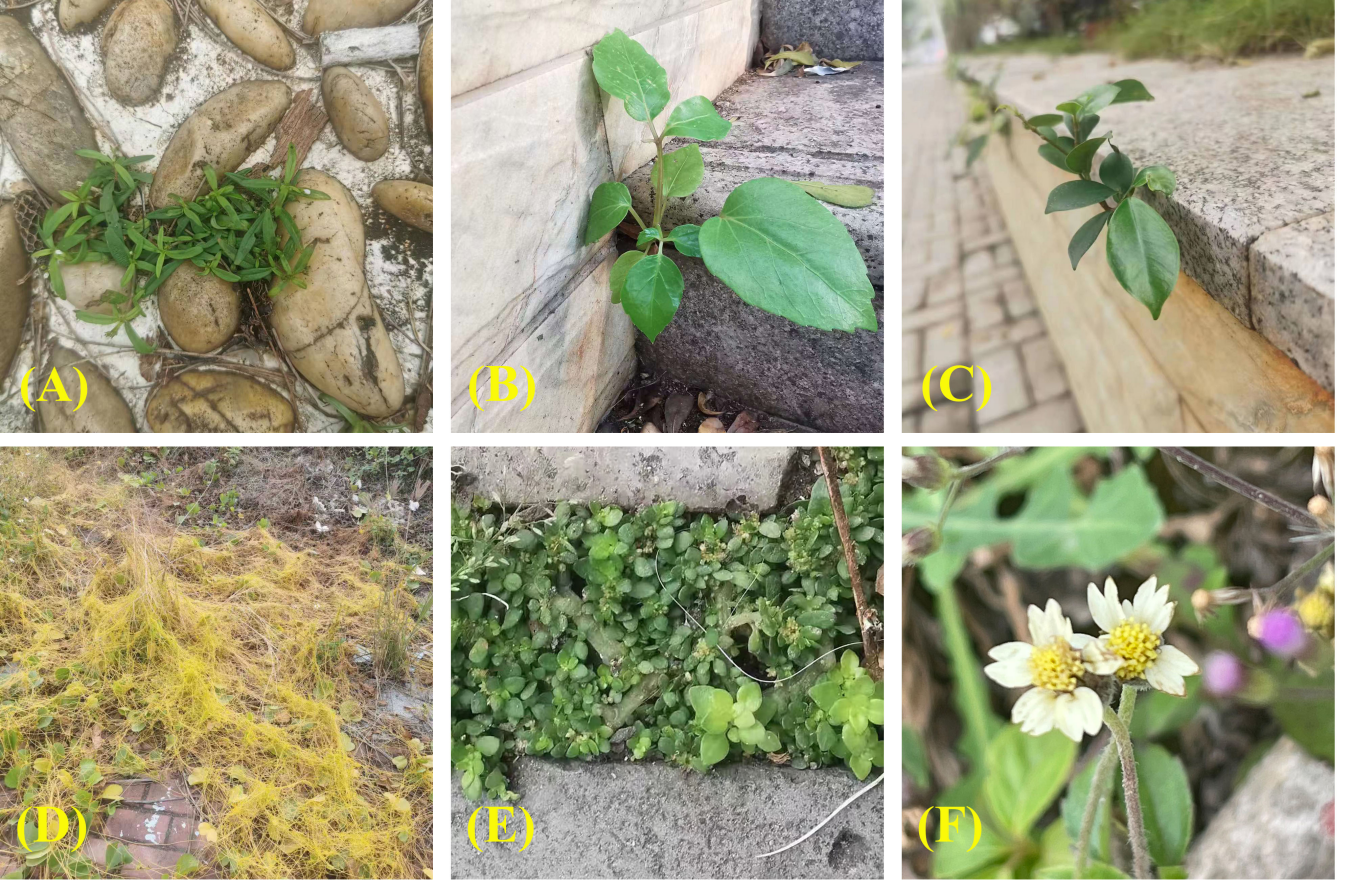
Examples of spontaneous species occurring within secondary urban functional units. **(A)** *Hedyotis corymbosa* (L.) Lam. **(B)** *Spathodea campanulata* P. Beauv. **(C)** *Ficus microcarpa Linn. f*. **(D)** *Cuscuta chinensis* Lam. **(E)** *Pilea microphylla* (L.) Liebm. **(F)** *Tridax procumbens* L.

After selecting the 111 different UFUs in Haikou City, a purpose-driven sampling method was used to establish one to three 20m × 20m tree sample plots in each UFU from June to October 2015 (Wang and López-Pujol, 2015). Similarly, a stratified sampling method was used to establish five 5 m × 5 m shrub samples and five 1 m × 1 m herb samples. These were taken at the four corners and the center of each 20 m × 20 m sample square, respectively. For UFU sample plots smaller than 20 m × 20 m, the Split Modified-Whittaker (SMW) sampling method was used for gathering UPD data (Barnett and Stohlgren, 2003). The identity and number of plant species within each UFU were recorded, with plant identifications based on land use, information provided by land use managers or homeowners, and the field staff’s botanical expertise. The Huabanlv software (http://hbl.nongbangzhu.cn/) was utilized to identify unknown plant species. All of the plants identified were classified as either cultivated species (**Figure 3**) or spontaneous species (**Figure 4**).

### 3.4 Land cover data

**Figure 5:**
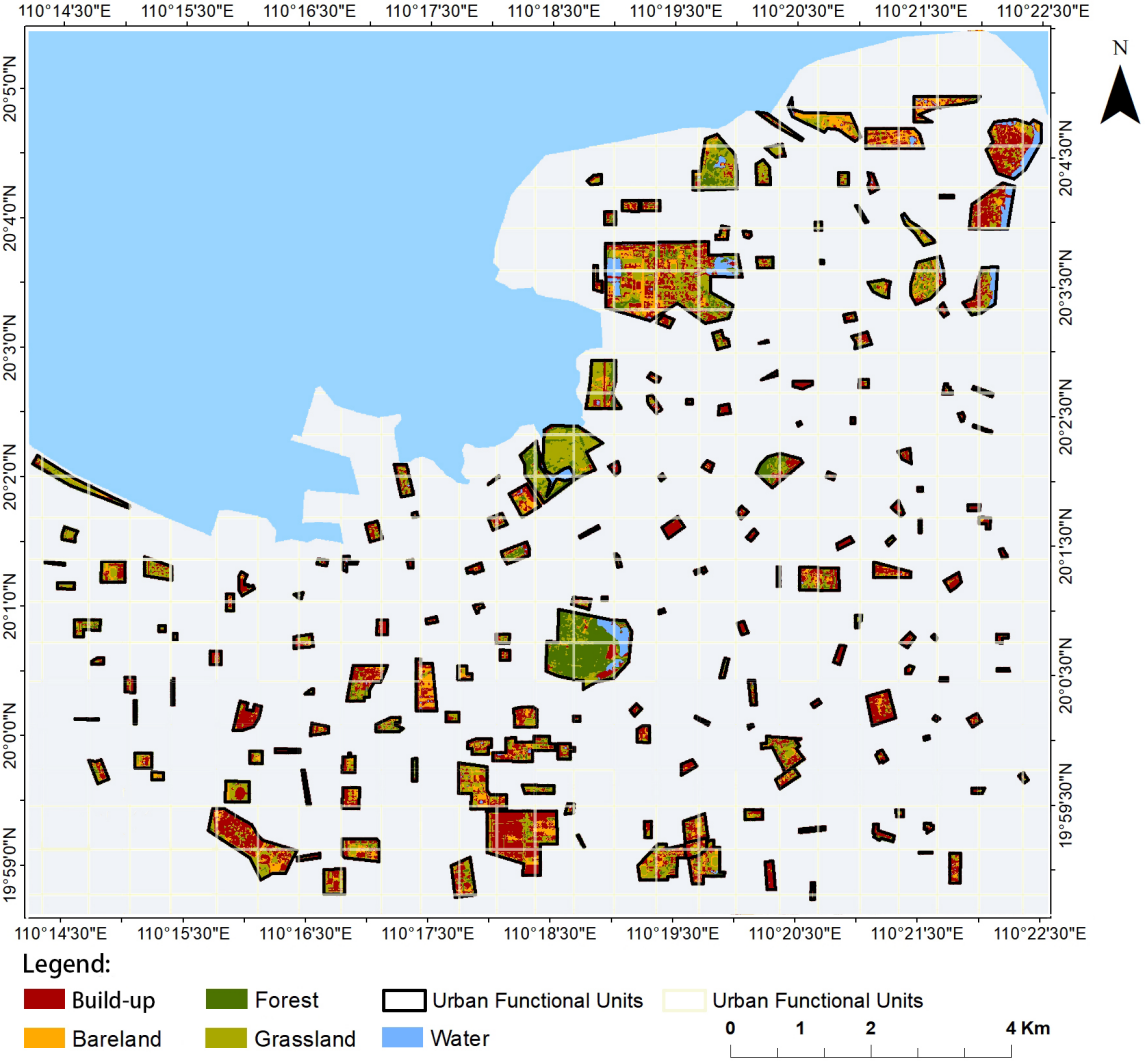
Land Cover (LC) data for the sampled Urban Functional Units (UFUs) in Haikou, China.

We acquired 30 m spatial resolution Landsat 5 images of Haikou City in 2015 from the U.S. Earth Resources Observation and Science (EROS) Center (https://www.usgs.gov/centers/eros). As Haikou city has low-density cloud coverage, we used the images from May to June 2015 to calculate urban land use classifications. The Landsat image preprocessing included cloud-free radiometric calibration, transient atmospheric correction, image extraction, image stitching and cropping, and vegetation index calculation. Based on Landsat 8 imagery in 2015, visual interpretation using ENVI 5.1 was performed to classify land use types in Haikou City, and the final land use types within 111 urban functional units in Haikou City were obtained. Following Zhang et al. (2022b), we divided land use types into five categories, which are classified and defined as follows: (1) Built-up areas (Bui) are land dominated by human constructions, in and around urban areas, including buildings and construction sites, (2) Tree and shrub area (Tre), which includes forested areas, native or non-native, consisting of tall trees with a dense canopy, (3) Herb area (Her), which contain predominantly herbaceous species, (4) Water, representing urban lakes and river water, and (5) Bare land, representing undeveloped land (**Figure 5**).

### 3.5 Socio-economic and management factors

We obtained the construction age (CA) of each UFU through Baidu and field interviews with building managers (Wang and López-Pujol, 2015). Housing price (HP) data were obtained through the official Anjuke website (https://beijing.anjuke.com/). Population density (PD) was obtained through field surveys of the population living in each urban functional unit, following Wang et al. (2016).

### 3.6 Greening management factors

These factors include the frequency of maintenance operations and conservation strategies. Management factors include the number of maintenance times per year (MT) (e.g., pruning and mowing), the number of watering times per year (WT), and the number of fertilizer application times per year (FT), which is likely to relate to other forms of management such as application of agrochemicals. The conservation management factors were identified by reviewing the records of each UFU management department, or if not feasible, by interviewing at least three management workers per plot.

### 3.7 Analytical methods

Boxplots visualizing the green space area and proportion, as well as the species richness (the number of total, cultivated, and spontaneous species richness) in each UFU category were drawn using “ggplot 2” in R (Wickham, 2016). IBM SPSS Statistics 23.0 was used to calculate Z-Score values for all variables to identify and remove outliers (defined as greater than 3, or less than -3) (Kreyszig, 1979). Multiple regression analysis was conducted to determine the combined and individual effects of the following potential UPD predictors of land cover [LC; including Urban green space (UGS) and Built-up area (Bui)], socio-economic [Soc; including Construction Age(CA), Housing Price(HP), and Population Density (PD)], and management variables [Man; including Maintenance times per year (MT), Fertilizing times per year (FT), and Watering times per year (WT)] on species diversity (the number of spontaneous (natural, unplanted species), cultivated, total, tree, shrub, and herb species). To detect multicollinearity, the variance inflation factor was used. This measures the correlation and strength of correlation between the predictor variables in a regression model. Multiple regression analysis and variance inflation factor analysis was performed in R 4.0.4 (https://cran.r-project.org/bin/windows/base/).

## 4 Results

### 4.1 Proportion of UGS in different UFUs

**Figure 6:**
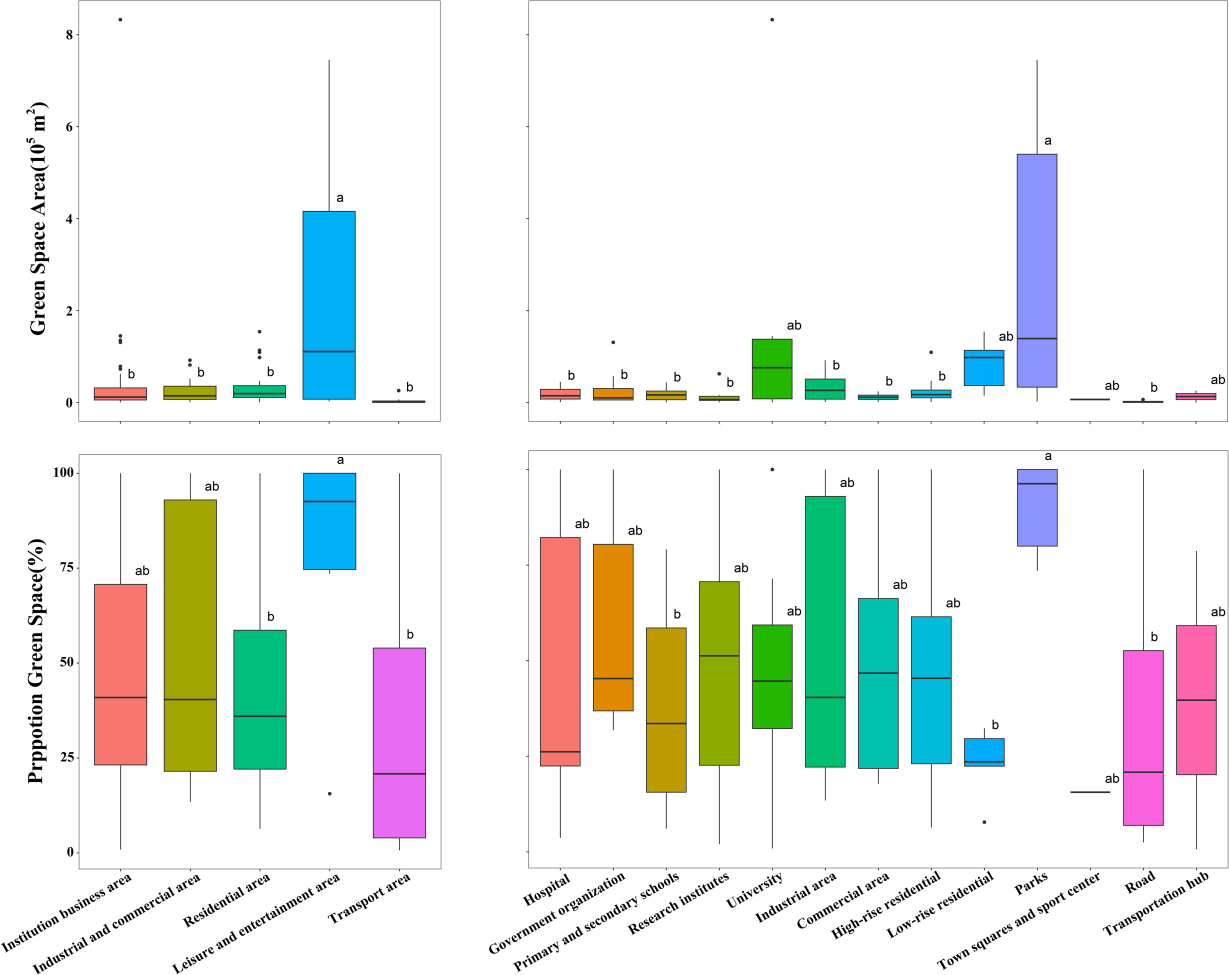
Total area and proportion of green space in each urban functional unit category. The lower case letters a and b indicate the results of Ýifferent group comparisons. A and B are different and differ significantly from each other.

In the primary UFUs, the highest UGS area is found in the leisure and entertainment area (243585 m^2^ ± 298080 m^2^), and the lowest is in the transport area (4248 m^2^ ± 7529 m^2^) (**Table 2**; **Figure 6**). Similarly, the highest percentage of UGS is in the leisure and entertainment area (79 ± 28%), and the lowest is in the transport area (34 ± 33%). Looking into the secondary UFU categories in more detail, the highest UGS area is parks (283056 m^2^ ± 304556 m^2^), and the lowest is roads (2030 m^2^ ± 2109 m^2^). The highest percentage of UGS is in parks (90 ± 11%), and the lowest is in sports centers (15 ± 0%).

**Table 2:** The mean and standard deviation of the area and percentage of green space found in each primary and secondary urban functional unit category.

Primary UFU	UGS area (m^2^)	UGS (%)	Secondary UFU	UGS area (m^2^)	UGS (%)	Number of plots
Institution business area	25203 ± 26178	53 ± 33	Hospital	19051 ± 15354	46 ± 38	6
			Government organization	30919 ± 41245	58 ± 26	8
			Primary and secondary schools	16673 ± 12533	38 ± 24	15
			Research institutes	15259 ± 19931	48 ± 33	7
			University	159593 ± 259955	45 ± 30	8
Industrial and commercial area	45348 ± 12597	45 ± 30	Industrial area	34931 ± 30296	55 ± 35	11
			Commercial area	11828 ± 7523	51 ± 36	8
Residential area	31858 ± 36435	42 ± 26	High-rise residential	21909 ± 21709	46 ± 27	26
			Low-rise residential	83595 ± 50876	23 ± 8	5
Leisure and entertainment area	243585 ± 298080	79 ± 28	Parks	283056 ± 304556	90 ± 11	6
			Town squares and sport left	6757 ± 0	15 ± 0	1
Transport area	4248 ± 7529	34 ± 33	Road	2030 ± 2109	32 ± 32	8
			Transportation hub	13118 ± 12933	39 ± 38	2

### 4.2 Distribution of species in different UFUs

**Figure 7:**
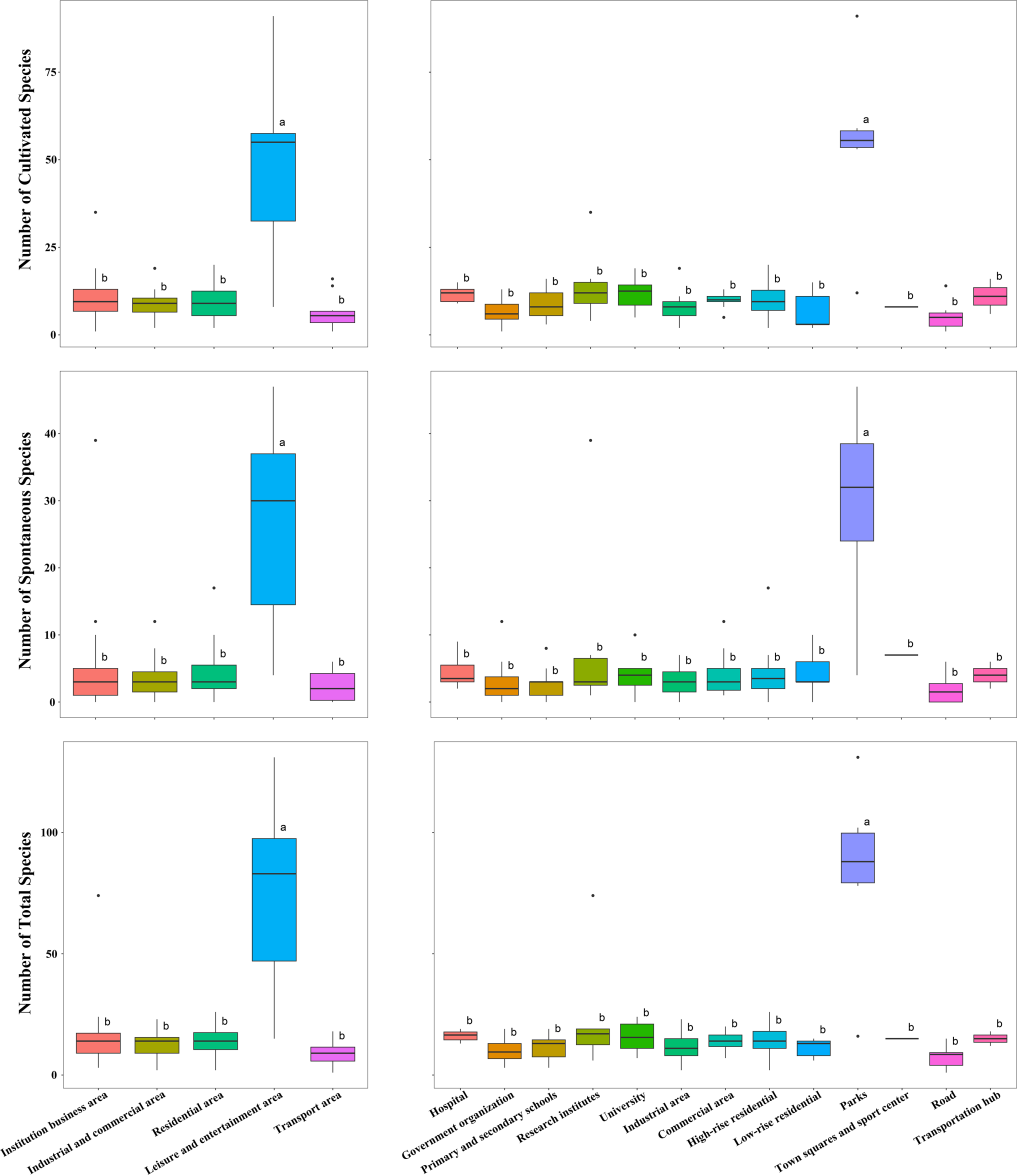
The number of spontaneous and cultivated species richness in each urban functional unit category. The lower case letters a and b indicate the results of Ýifferent group comparisons. A and B are different and differ significantly from each other.

In the primary UFUs, the highest total species richness is found in leisure and entertainment areas (72 ± 39), while the lowest is in transport areas (8 ± 5) (**Table 3**; **Figure 7**). Similarly, the highest spontaneous species richness is in leisure and entertainment areas (26 ± 15), and the lowest is in transport areas (2 ± 2). The greatest cultivated species richness is also seen in leisure and entertainment areas (47 ± 26), while the lowest is in transport area (6 ± 5).

**Table 3:** The mean and standard deviation of the area and percentage of green space in each urban functional unit category.

Primary UFU	Total species richness	Number of spontaneous species richness	Number of cultivated species richness	Secondary UFU	Total species richness	Number of spontaneous species richness	Number of cultivated species richness
Institution business area	14 ± 10	4 ± 6	10 ± 6	Hospital	14 ± 2	5 ± 2	11 ± 2
				Government organization	10 ± 5	3 ± 4	7 ± 4
				Primary and secondary schools	11 ± 4	3 ± 2	9 ± 4
				Research institutes	22 ± 21	8 ± 12	14 ± 9
				University	15 ± 6	12 ± 4	11 ± 4
Industrial and commercial area	12 ± 5	4 ± 3	9 ± 4	Industrial area	10 ± 6	3 ± 2	8 ± 4
				Commercial area	13 ± 4	4 ± 4	10 ± 2
Residential area	13 ± 5	4 ± 3	10 ± 5	High-rise residential	13 ± 5	4 ± 3	10 ± 5
				Low-rise residential	11 ± 4	4 ± 3	7 ± 5
Leisure and entertainment area	72 ± 39	26 ± 15	47 ± 26	Parks	81 ± 33	30 ± 14	54 ± 23
				Square and sport left	15 ± 0	7 ± 0	8 ± 0
Transport area	8 ± 5	2 ± 2	6 ± 5	Road	7 ± 4	2 ± 2	5 ± 4
				Transportation hub	14 ± 4	4 ± 2	11 ± 5

In the secondary UFUs, the highest total species richness is found in parks (81 ± 33), and the lowest is in roads (7 ± 4) (**Table 3**; **Figure 7**). The most spontaneous species richness is in parks (30 ± 14), and the lowest is in roads (2 ± 2). The most cultivated species richness is in parks (54 ± 23), and the least is in roads (5 ± 4).

Within the institutional business area category, research institutes show the highest total species richness, with a significant portion being spontaneous species, while government organizations have the least. Commercial areas show slightly higher species richness than industrial ones, while both high-rise and low-rise residential areas have similar species richness. Within the leisure and entertainment area category, parks were found to contain significantly higher total, spontaneous, and cultivated species richness than squares and sports centers. Finally, transportation hubs have higher numbers of total, spontaneous, and cultivated species richness than areas dominated by roads (**Figure 7**).

### 4.3 Assessment of potential driver attributes on different numbers of species

The results of the multiple linear regression (**Table 4**) show that UGS has a positive effect on the number of spontaneous species (SpS) (β = 0.393*) and herb species (HS) (β = 0.338*). However, the β-coefficients indicate that UGS does not significantly impact ToS, CS, TrS, or ShS. We also find that maintenance times (MT) have a positive effect on the number of total species (ToS) (β = 0.105*), the number of cultivated species (CS) (β = 0.106*), tree species (TrS) (β = 0.106*), and HS (β = 0.132*). Similarly, the results show that watering frequency (WT) has a positive effect on CS (β = 0.098*) and ShS (β = 0.185***). However, construction age (CA) has a negative effect on the number of shrub species (ShS) (β = -0.151*). Built-up areas do not significantly affect any outcome variables based on the β-coefficients, as shown. Similarly, HP, PD and FT did not show a significant impact on any of the outcome variables (**Table 4**).

**Table 4:** Multiple regression analysis showing the effects of urban green space (UGS), built-up area (Bui), construction age (CA), housing price (HP), population density (PD), maintenance times (MT), fertilizing times (FT), and watering frequency (WT) on the number of total species (ToS), spontaneous species (SpS), cultivated species (CS), tree species (TrS), shrub species (ShS), and herb species (HS).

Different factors	ToS (R^2 =^ 0.289)	SpS (R^2 =^ 0.038)	CS (R^2 =^ 0.334)	TrS (R^2 =^ 0.200)	ShS (R^2 =^ 0.353)	HS (R^2 =^ 0.092)
	β-coefficient	β-coefficient	β-coefficient	β-coefficient	β-coefficient	β-coefficient
Intercept	-0.170***	-0.171***	-0.158***	-0.185***	-0.143***	-0.153***
UGS	0.089	0.393*	-0.118	-0.198	0.032	0.338*
Bui	0.079	0.014	0.100	0.128.	0.082	0.017
CA	**-0.098.**	-0.145.	-0.053	-0.038	-0.151*	-0.071
HP	0.029	0.011	0.008	-0.037	0.106	0.010
PD	-0.066	-0.106	-0.020	-0.025	-0.029	-0.111
MT	0.105*	**0.103.**	0.106*	0.118*	**0.022.**	0.132*
FT	0.115	0.026	0.159	0.170.	0.154	0.023
WT	0.045	-0.059	0.098*	-0.001	0.185***	-0.034

The asterisks show significance levels: ‘***’ <0.001, ‘**’ <0.01, ‘*’ <0.05.

Bold values represent data of significance.

The variance inflation factors of each multiple regression model predictor variable (ToS, SpS, CS, TrS, ShS, HS) ranged between 1.073 to 2.400 (**Table 5**), indicating low to moderate correlation between a given predictor variable and other predictor variables in the model. This was not considered significant enough to influence the model reliability. This indicates that the predictor variables used in the regression models are reasonably independent and can be used in multiple linear regression analysis without any covariance issues.

**Table 5:** Variance Inflation Factors of each multiple regression of the number of total species (ToS), spontaneous species (SpS), cultivated species (CS), tree (TrS) species, shrub species (ShS), and herb species (HS) with urban green space (UGS), built-up area (Bui), construction age (CA), housing price (HP), population density (PD), maintenance times (MT), fertilizing times (MT), and watering frequency (WT).

Different factors	ToS	SpS	CS	TrS	ShS	HS
UGS	1.956	1.956	1.956	1.956	1.956	1.956
Bui	2.122	2.122	2.122	2.122	2.122	2.122
CA	1.306	1.306	1.306	1.306	1.306	1.306
HP	1.073	1.073	1.073	1.073	1.073	1.073
PD	1.180	1.180	1.180	1.180	1.180	1.180
MT	2.400	2.400	2.400	2.400	2.400	2.400
FT	2.252	2.252	2.252	2.252	2.252	2.252
WT	1.964	1.964	1.964	1.964	1.964	1.964

## 5 Discussion

### 5.1 Distribution of UGS in different UFUs

Leisure and entertainment areas, including parks and gardens, are often designed with UGS to increase the comfort and enjoyment of visitors. Conversely, transport areas are primarily designed for transportation purposes and tend to have less UGS. This observation aligns with previous research indicating that UGS tends to be concentrated in specific city areas like parks and recreation zones, while other zones, such as industrial or commercial ones, are often deficient in green spaces (Zhu et al., 2019; Zhang et al., 2022a). The presence of UGS in urban areas is crucial for various reasons. They offer numerous environmental benefits, such as improved air quality, reduced urban heat island effects, and climate change mitigation (Latinopoulos, 2022). They also provide social benefits, such as promoting physical activity, improving mental health, and facilitating social interaction (Nutsford et al., 2013; Jennings and Bamkole, 2019). Therefore, it is critical for urban planners and policymakers to consider the distribution of UGS in cities, ensuring that all residents can access these benefits, irrespective of their locality (Lee et al., 2015). Criteria for selecting UGS sites should include visual, recreational, and aesthetic considerations, alongside ecological and open space functionalities (Panagopoulos et al., 2016). By increasing the number and improving the design of parks and recreational areas, UGS can play a pivotal role in promoting sustainability and guiding urban development toward a balanced and harmonious relationship with nature (Anguluri and Narayanan, 2017; Douglas et al., 2017). Finally, planners and policymakers need to consider the long-term costs and benefits of UGS development, paying special attention to the role that plant biodiversity can play in supporting the stability and resilience of urban ecosystems.

### 5.2 Distribution of species across different UFUs

In this study, the highest numbers of cultivated, spontaneous, and total species were observed in leisure and entertainment areas. Because most leisure and entertainment areas are open to the public (Ramer and Nelson, 2020), this implies a significant governmental investment in maintaining these areas, which may outweigh investments in other UFUs, such as transport areas (Furlan and Sinclair, 2021). As a consequence, this higher level of investment appears to pay dividends in more than just species richness. The lower species richness observed in UFUs aside from leisure and entertainment areas may be due to a lower investment in the ecological planning, management and maintenance of these areas. Hence there is considerable potential to improve the species richness of UFUs which traditionally possess lower species richness, with a view toward more sustainable urban development. For example, planners may opt for small-statured species in residential, business and transport UFUs for aesthetic and practical reasons (Threlfall et al., 2016).

In the secondary UFUs, the highest total species richness is seen in parklands, and the lowest is in roads. These results suggest that the total species richness in secondary UFU types varies depending on the location, which can be attributed to several factors. Firstly, parks are typically designed and managed to preserve natural habitats and promote biodiversity, explaining the higher species richness typically observed in these areas (Alvey, 2006). Parks often contain a diverse array of vegetation, including native trees, shrubs, and flowers, which can support a wider variety of animal species, including birds, insects, and small mammals (Idilfitri and Mohamad, 2012). Additionally, parks are more likely to provide a more suitable habitat for species with particular habitat requirements, such as large contiguous forest areas.

On the other hand, roads and similar areas with a lower UPD tend to be fragmented and disconnected, making them less hospitable for many species (Liu et al., 2019). Roads are often characterized by a lack of vegetation and high levels of anthropogenic disturbance, which impacts negatively on the local ecology (Zhang et al., 2022a). Road construction and maintenance activities can contaminate soils or destroy natural habitats, while the constant flow of traffic and associated pollution produced can further detract from the species diversity (Ukaogo et al., 2020). Additionally, roads can create physical barriers that impede animal movement and plant dispersal, limiting their ability to establish new populations.

The distribution of cultivated plant species follows a similar trend to the total species richness, peaking in leisure and entertainment areas and declining around roads. Like gardens and parks, leisure areas feature a diverse range of ornamental species, bolstered by suitable habitats with ample nutrients, irrigation, and sunlight (Middle et al., 2014). As previously mentioned, roads and urbanized areas are less hospitable to plant cultivation, with constant traffic, pollution, poorer soil conditions, and maintenance treatments such as herbicide application (Nicholls and Altieri, 2013). In residential areas, both high-rise and low-rise developed show similar species richness, likely due to the presence of green spaces. Despite greatly differing building and population densities, intentionally planned green spaces can maintain biodiversity in a range of residential spaces.

Finally, amongst institutional and commercial UFUs, research institutes contain the highest species richness, which may be attributed to the presence of botanical gardens, arboretums, and sustainable landscaping practices. In contrast, government organizations show the lowest diversity. These are often located in highly urbanized centers with limited green spaces; however, there is certainly potential to better manage UGS and plant choices in these organizations to enhance urban biodiversity. Commercial primary UFUs tend to show a slightly higher species richness over industrial areas, likely due to more green spaces. Industrial areas, which almost inevitably prioritize functionality over aesthetics, have very few green spaces and subsequently low species richness. Integrating more urban green spaces in industrial areas is important to increase biodiversity; however, uptake of green spaces in these localities is likely to require demonstration of a clear benefit to the company.

Overall, our results highlight the importance of preserving and promoting natural habitats in urban areas, particularly in areas such as parks, where biodiversity is naturally the highest. The results also underscore the need for careful planning and management of all aspects of urban infrastructure, including roads, to minimize their impact on local ecosystems.

### 5.3 Potential drivers for urban plant diversity

UGS offer numerous benefits to humans and play a crucial role in conserving UPD. However, urban biodiversity can be threatened by common UGS management practices, such as turf lawn maintenance, pesticide and herbicide applications, and the introduction of non-native plant species. In addition, socioeconomic and cultural dynamics managed by multiple stakeholders significantly influence urban biodiversity. Our study demonstrates that UGS is positively correlated with the number of shrub species and herb species, indicating that UGS presence can enhance biodiversity and ecosystem services by fostering the growth and development of diverse plant species in the urban environment. UGS provides suitable habitats, food sources, and shelter for a range of species, thereby contributing to the overall biodiversity of the area.

Construction age was found to negatively affect ShS, meaning that the number of shrub species declines as the age of UGS construction increases. This relationship between UGS construction age and ShS may be due to several factors, such as changes in soil conditions (Uno et al., 2010), alterations to natural water flow patterns (Rupprecht et al., 2015), and the removal of native vegetation during the construction process. The UGS construction disrupts the surrounding area’s soil and natural drainage patterns, making it more difficult for shrub species to establish and thrive. In addition, construction processes often involve the removal of native vegetation, which further reduces the number of shrub species in the area. It is feasible that older UGS had less attention paid to their ecological impact during the construction process, leading to greater disruption of the surrounding environment. Over time, as environmental regulations and awareness increase, newer UGS tends to be designed with greater consideration for their impact on surrounding ecosystems. Overall, our findings suggest that UGS construction has a negative impact on shrub species and that efforts should be made to mitigate this impact through careful planning and implementation of construction projects. Additionally, our study highlights the importance of considering the long-term ecological impacts of urban development on green spaces and natural ecosystems.

Maintenance times were found to positively impact the number of total species, cultivated species, tree species, and herb species in urban green spaces, indicating that regular maintenance can enhance overall species richness and diversity. MT activities such as pruning can lead to healthier and more diverse plant populations, promoting plant growth and survival. Additionally, regular maintenance can help control invasive species, which have well-documented, adverse effects on native plant communities, reducing species diversity (Shackleton et al., 2019). A further way in which maintenance activities can promote species diversity is through creating suitable habitats for certain species, such as ground- dwelling insects or birds. The significance of regular maintenance practices in urban areas highlights the need for sustainable maintenance practices to support plant health and conservation.

Watering frequency also positively affects the diversity of cultivated and shrub species, highlighting the importance of proper water management in urban green spaces. Adequate water supply is important for supporting plant growth, increasing flowering and fruiting rates, and improving plant health (Rosa et al., 2020). In particular, many cultivated species require regular watering to maintain their ornamental value and aesthetic appeal (Pardossi et al., 2009). Additionally, while shrub species tend to be more drought-tolerant than other plant types, they can still benefit from regular watering, especially during periods of prolonged drought (Liu et al., 2012). Moreover, maintenance of higher soil moisture levels can have positive impacts on soil microorganisms and other soil-dwelling organisms, like earthworms. These organisms contribute to improved soil quality, nutrient availability, and overall plant health, resulting in a more diverse ecosystem. Consequently, suitable watering regimes are essential for plant growth and conservation, particularly in urban areas with limited water resources. This also highlights the need for sustainable water management practices, such as using recycled or harvested rainwater, to minimize water waste while promoting plant diversity. In short, this study indicates that regular maintenance and suitable watering regimes can enhance the growth and development of different plant species in urban environments. This information is also crucial in assisting urban planners and policymakers to create more sustainable green urban areas that support biodiversity and ecosystem services, as it highlights that maintaining biodiversity is an ongoing process. Planning and budgeting for UGS should be conducted accordingly.

Other factors such as UGS, built-up areas, housing prices, population density, and fertilizing frequency have limited influence on the number and variety of plant species in urban environments (e.g., total species, cultivated species, tree species, and shrub species). This implies that other factors, such as specific types of human intervention, local climate, or soil type, may play a more important impact on urban biodiversity. However, these findings do not entirely dismiss the effects of the studied factors; their influence may be subtle, indirect, or mediated by other variables not considered in this analysis. Thus, more comprehensive studies incorporating various environmental, social, and economic variables are required to better to comprehend the drivers of biodiversity in urban green spaces. Ultimately, this study suggests that regular maintenance and proper watering can enhance the growth and development of plant species in urban environments, which can inform urban planners and policymakers in creating sustainable green areas which support biodiversity and ecosystem services.

### 5.4 Future research directions

An in-depth analysis of the UGS distribution showed significant variation between UFUs, so future research needs to provide more comprehensive data collection in regions with smaller samples. Spatial availability, policy frameworks, and public perceptions about urban green spaces will help develop targeted solutions for expanding UGS (Kabisch et al., 2016). Researchers could explore innovative strategies like vertical gardens, green roofs, or pocket parks in industrial or commercial zones, including transforming abandoned lots into green spaces (Lehmann, 2014). Other work is needed to assess the impact of native plant species, wildlife habitats, and biodiversity-friendly maintenance practices which may promote urban biodiversity (Plummer et al., 2020). Additionally, investigating the role of community engagement through gardens or citizen science projects is crucial. Despite the non-significant relationships with socio-economic factors seen in this study, understanding UGS’s contribution to social and economic well-being is vital for informing policies and investments in urban green infrastructure. Considering cities’ role in climate change, studying the effect of UGS on cooling urban heat islands, flood mitigation, and carbon sequestration is another essential research topic. Addressing these research directions will support effective UGS implementation and management, fostering urban sustainability and biodiversity conservation into the future.

## 6 Conclusions

UGS and UPD play crucial roles in improving the quality of life for city dwellers. This study examined the distribution of UGS and UPD in various UFUs in Haikou City, demonstrating that leisure and entertainment areas – such as parks and recreational spaces – had the highest UGS coverage and species richness, while transport areas showed the lowest. Additionally, the study assessed potential drivers of UPD, demonstrating a positive correlation between UGS area and the number of spontaneous species and herb species. In contrast, construction age negatively impacts on the number of shrub species. The findings underscore the importance of proper planning and investment in UGS to encourage sustainability and guide urban development toward a more balanced and harmonious relationship with nature. These results can provide valuable insights for policymakers and urban planners with respect to the significance of UGS distribution and biodiversity enhancement in urban environments. This paper lays the groundwork for city managers to understand the benefits and services of UGS and UPD and to consider the rational allocation of UGS and UPD across different UFUs in urban planning and management.

## Data availability statement

The raw data supporting the conclusions of this article will be made available by the authors, without undue reservation.

## Author contributions

Conceptualization, H-LZ, L-YG, MN, and H-FW. Methodology, H-LZ, L-YG, MN, H-FW. Software, H-LZ, L-YG, MN, and H-FW. Validation, H-FW. Formal analysis, H-LZ and MN. Investigation, H-FW. Resources, H-FW. Data curation, H-FW. Writing—original draft preparation, H-LZ, L-YG, MN, and H-FW. Writing—review and editing, H-LZ, L-YG, MN, and H-FW. Visualization, H-FW. Supervision, H-FW. Project administration, H-FW. Funding acquisition, H-FW.
